# Examination of the validity of the Chinese version of the Language Mindsets Inventory among Chinese university students

**DOI:** 10.3389/fpsyg.2024.1437164

**Published:** 2024-09-16

**Authors:** Pengpeng Cai, Xuhong Li, Zheyuan Mai, Juxiong Feng, Xin Guan

**Affiliations:** ^1^Trinity Centre for Global Health, School of Psychology, Trinity College Dublin, Dublin, Ireland; ^2^Department of Social and Behavioural Sciences, City University of Hong Kong, Kowloon, Hong Kong SAR, China; ^3^Department of Social Work, Hong Kong Baptist University, Kowloon, Hong Kong SAR, China

**Keywords:** language mindset, growth mindset, fixed mindset, Chinese university students, validation, confirmatory factor analyses

## Abstract

**Introduction:**

With the growing interest in the psychological basis of language learning, this study aims to validate the Chinese version of the Language Mindset Inventory (LMI) among Chinese university students.

**Methods:**

A sample of 476 students from various universities in mainland China was used. The translation process followed the forward-backward method. Confirmatory factor analyses (CFAs) were conducted to evaluate the factor structure. The internal consistency of the LMI was assessed using Cronbach's alpha, and convergent validity was examined through correlations with established mindset measures.

**Results:**

Compared with the one-factor model, the two-factor model which distinguishes between fixed and growth mindsets, showed an acceptable and better fit index. The overall Cronbach's alpha for the full scale was 0.954, with 0.929 for the fixed mindset dimension and 0.903 for the growth mindset dimension. AVE values for the fixed and growth dimensions were 0.597 and 0.510, respectively. Correlations showed that the growth mindset dimension was significantly positively associated with the 8-Item Growth Mindset Scale (*r* = 0.186, *p* < 0.01) and the Mindset Scale for Learning English (*r* = 0.149, *p* < 0.01), while the fixed mindset dimension was negatively associated with both scales (*r* = −0.228 and *r* = −0.169, *p* < 0.01).

**Discussion:**

This study confirms the reliability and validity of the Chinese version of the LMI, making it a relevant tool for assessing language learning mindsets among Chinese university students. The findings support the integration of LMI into educational strategies to promote resilience and adaptability, enhancing language education outcomes.

## Introduction

Mastery of a second language is increasingly recognized as an essential element of global competence, especially in higher education (Lasagabaster, 2022; Zhai and Wibowo, 2023). For university students, this skill not only bridges cultural and communication gaps, but also facilitates their integration into the global community. In China, where English is often a second language, the development of language skills is critical to students' future career paths and personal growth (Zhang et al., 2020). Therefore, it is imperative to prioritize English education at the university level to prepare students for the demands of a globalized world.

Within educational psychology, the concept of “language mindset” has significantly advanced the understanding of how beliefs about the malleability of language learning ability influence student outcomes. Originating from the broader framework of mindset theory proposed by Molden and Dweck (2006), language mindset categorizes beliefs into two core attitudes: “fixed” and “growth.” Individuals with a fixed mindset view intelligence as stable and unchangeable, while those with a growth mindset believe in its malleability and the potential for improvement through effort (Dweck, 2017; Dweck and Yeager, 2020; Hallahan, 2020).

Building on the foundational concepts of mindset theory articulated by Molden and Dweck (2006), subsequent research has endeavored to integrate these principles into the field of language learning. The seminal study by Lou and Noels (2016) substantially enhanced the comprehension of this field by investigating the correlations between particular beliefs about language proficiency, referred to as “language mindset,” and their consequent effects on language learning outcomes Their “mindset-goal-response” model further illustrates how language mindset influences goal orientation in language learning and responses to challenging situations (Lou and Noels, 2016, 2019; Tapia Castillo, 2023). Learners with a growth mindset pursue learning goals that lead to mastery responses and low anxiety levels, whereas learners with a fixed mindset pursue performance goals that result in helplessness responses and increased anxiety when faced with challenges (Lou and Noels, 2016, 2017). Furthermore, empirical studies have shown that students who adopt a growth mindset in language learning are more likely to persevere through challenges and achieve higher levels of performance (Lou et al., 2022; Sadeghi et al., 2020; Zhao et al., 2018). Thus, the concept of language mindset not only enriches the understanding of the psychological underpinnings of language acquisition but also underscores the potential of fostering a growth mindset to improve learners' language learning outcomes.

Following the theoretical developments, there has been a growing interest in exploring the impact of language mindsets on language education outcomes (Zarrinabadi and Lou, 2022). Recent research has increasingly studied the relationship between language mindset and various outcomes of language learning as well as other psychological factors, illustrating the profound impact of mindset about language on learner behavior and performance (Bai and Wang, 2023; Khajavy et al., 2021). For example, Ozdemir and Papi's (2022) study suggests that learners with a fixed mindset experience higher level of speaking anxiety, while those with a growth mindset show greater confidence in their language abilities. Similarly, Lou and Noels (2020) found that growth mindset learners are more resilient and open to intercultural communication, while fixed mindset learners tend to avoid such interactions due to fear of rejection. These findings underscore the importance of fostering a growth mindset to enhance both language proficiency and social engagement in educational settings.

Extending this research within the Chinese context, studies like Yao et al. (2021) observed that growth-mindset learners among Chinese students are more likely to perceive themselves as competent and respond to learning challenges with mastery-oriented behaviors. Furthermore, Hu et al. (2022) have demonstrated that Chinese university students with growth mindsets not only achieve higher in English due to increased grit and enjoyment but also exhibit better adaptation to the challenges of learning English. However, these studies have used diverse methods to measure language mindset and using varied methods to measure language mindset complicates comparisons across studies, potentially obscuring key insights. To address this, this study aims to develop and validate a standardized Language Mindset Inventory tailored for Chinese university students, ensuring consistent and culturally relevant assessments.

The Language Mindset Inventory (LMI) is an essential tool used to assess students' language mindset empirically. The Language Mindset Inventory was developed by Lou and Noels (2017) based on Dweck's foundational mindset theory, further integrating Ryan and Mercer (2012) findings on motivation for language learning. The questionnaire is designed to measure individuals' beliefs about the plasticity of language ability, distinguishing between fixed and growth mindsets. Lou and Noels (2019) validation of the LMI demonstrated its robustness across diverse educational contexts, affirming its efficacy in accurately capturing learners' perceptions of growth potential in language learning.

Several studies have emphasized the indispensable role played by validated instruments such as the LMI. For example, researchers such as Zarrinabadi et al. (2021) and Khajavy et al. (2021) have used the LMI to effectively measure language mindset, thereby enabling educators to develop educational strategies to optimize language outcomes while addressing psychological aspects of language learning in different educational settings. Additionally, studies such as the one conducted in a Japanese EFL setting support the applicability of the LMI across cultures, demonstrating its robustness across educational settings (Collett and Berg, 2020). This international adaptability emphasizes the potential for LMI to be used and adapted effectively in other non-Western settings such as China. Despite the proven international utility of LMI and its successful application in countries, there has been little application in China, especially for Chinese university education. Existing research highlights the impact of LMI on Chinese students' language learning outcomes, yet the need for localized validation and adaptation of LMI to Chinese educational settings is evident. To address this gap, this study aims to develop and validate a Chinese version of the LMI to better match the specific needs and cultural differences of Chinese university learners.

With a sample of Chinese university students, this study rigorously evaluated the psychometric properties of the LMI by employing various analytical techniques. The examination encompassed (a) the factor structure, which we investigated using confirmatory factor analysis (CFA) techniques to validate both one-factor and two-factor models. (b) Assessed the internal consistency of the LMI, which was further confirmed by strong Cronbach's alpha values for the overall scale and each dimension and evaluated its convergent validity through the average variance extracted (AVE) criteria. External relations were examined through correlations with related measures such as the 8-Item Growth Mindset Scale and the Mindset Scale for Learning English.

## Method

### Participants

A development sample comprised of non-native English-speaking undergraduate students was utilized for the study. Participants were recruited from colleges or universities in mainland China. All procedures in this study were conducted in accordance with the ethical standards of the Institutional Review Board of University. Informed consent was obtained from all participants before the study, ensuring that they voluntarily participated after fully understanding the purpose, procedures, potential benefits, and risks associated with the research.

All measures were administered in the students' primary language, Mandarin. The questionnaires were translated into Chinese using the forward-backward translation method (Erkut, 2010). Two experts with professional backgrounds in English conducted the forward translation, and two experts with qualifications in Teaching Chinese as a Foreign Language performed the back translation. The research team reviewed and consolidated all translation results, making final decisions. Challenges included selecting appropriate Chinese equivalents for certain English terms, which required careful consideration due to subtle differences in meaning. Discrepancies and inconsistencies were resolved through consensus in collaborative meetings, ensuring the accuracy and consistency of the translations.

### Measurements

#### 8-Item Growth Mindset Scale

The 8-Item Growth Mindset Scale (GMS), developed initially by Dweck (1999), is the predominant brief scale used in applied research to measure mindset (Kern et al., 2015; Zhou et al., 2020). It demonstrates good internal consistency, with a Cronbach's alpha typically ranging between 0.70 and 0.90, indicating high inter-item correlations. Further validation in a study by Midkiff et al. (2018) confirmed the scale's reliability, with a Cronbach's alpha of 0.93, underscoring its robustness in assessing growth mindset. Responses are recorded on a 5-point Likert scale from 1 (strongly disagree) to 5 (strongly agree), with consistent response options across the five surveys comprising the sample. In the study conducted by the Midkiff et al. (2018), the internal consistency of the scale was evaluated using Cronbach's alpha. The obtained alpha value of 0.93 indicated good reliability.

#### Mindset Scale for Learning English

The Mindset Scale for Learning English (MSLE) was developed to assess learners' mindset orientations toward English learning, this tool comprises 10 items scored on a 4-point Likert scale, ranging from 1 (strongly agree) to 4 (strongly disagree). Adapted from Dweck (2006), it is designed to measure the continuum between fixed and growth mindsets. Total scores were determined. This scale indicates that the higher the score, the stronger the growth mindset in learning English.

#### Language Mindset Inventory

The study employed the complete Language Mindset Inventory (LMI), which includes 18 items designed to measure beliefs about the fixedness and growth potential of language abilities (Lou and Noels, 2017). Recent studies have validated the application of this scale, confirming its effectiveness in exploring fixed and growth mindset orientations in language learning (Hu et al., 2022; Nguyen, 2023).

### Data analysis

Data analysis was conducted using SPSS.26 and Mplus 8.3. Descriptive statistics were performed to illustrate participants' demographic information. The psychometric properties of the LMI were tested by construct validity and reliability. The structural validity of the one-factor structural model and two-factor structural model were computed by employing confirmatory factor analysis (CFA) in total sample and in both gender groups. The goodness of fit was accessed by using the following indices: comparative fit index (CFI > 0.90), Tucker Lewis index (TLI > 0.90), root-mean-square error of approximation (RMSEA <0.08) (Browne and Cudeck, 1992; Waltz et al., 2010). Additional, Hu and Bentler (1999) characterized RMSEA values from less than 0.08 up to 0.10 as “mediocre,” indicating a lower, yet acceptable, level of fit adequacy.

Regarding convergent and discriminant validity, we test the average variance extracted (AVE), with a cut-off value of 0.5 being the adequate level for convergent validity. Concurrent validity was assessed using Pearson's correlation coefficients between the LMI and 8-Item Growth Mindset Scale and Mindset Scale for Learning English. To be specific, Cronbach's alpha of 0.70 was viewed as an acceptable or acceptable level of reliability, while the acceptable CR value should be higher than 0.7 (Nunnally, 1994; Carmines and McIver, 1981).

## Results

### Characteristics of participants

In total, 476 university students completed the survey. The majority of them were female students (61.3%), and the mean age was 21.95 years (SD = 3.34). The mean scores of the sub-dimension were 3.03 (SD = 1.2) for fixed mindset and 4.10 (SD = 1.09) for growth mindset. The mean scores of each single item within LMI were between 2.67 (SD = 1.45) and 3.21 (SD = 1.46).

### Internal structure

**Figure 1:**
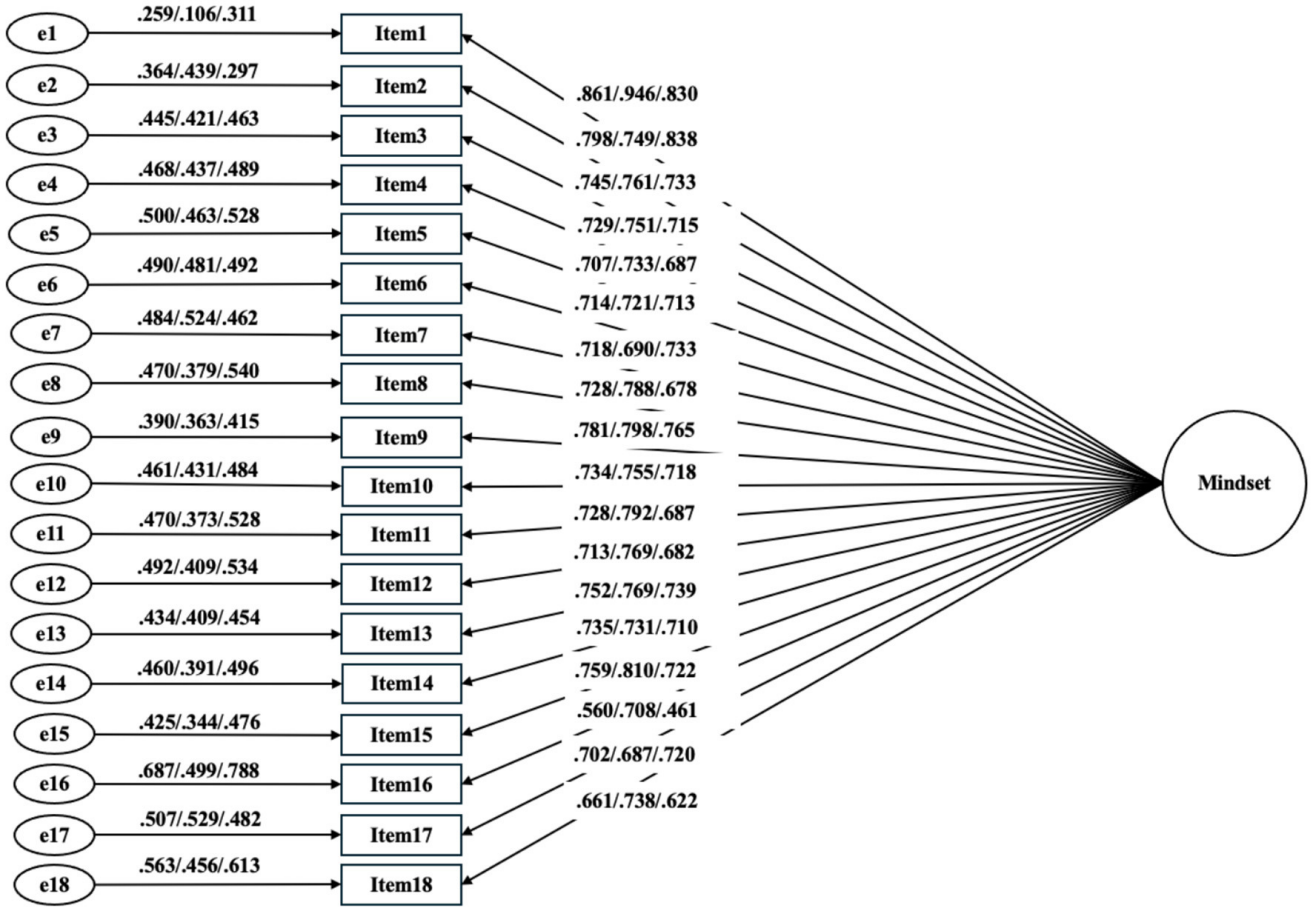
Standardized parameter estimates for the one-factor structure of the LMI among Chinese University Students.

**Figure 2:**
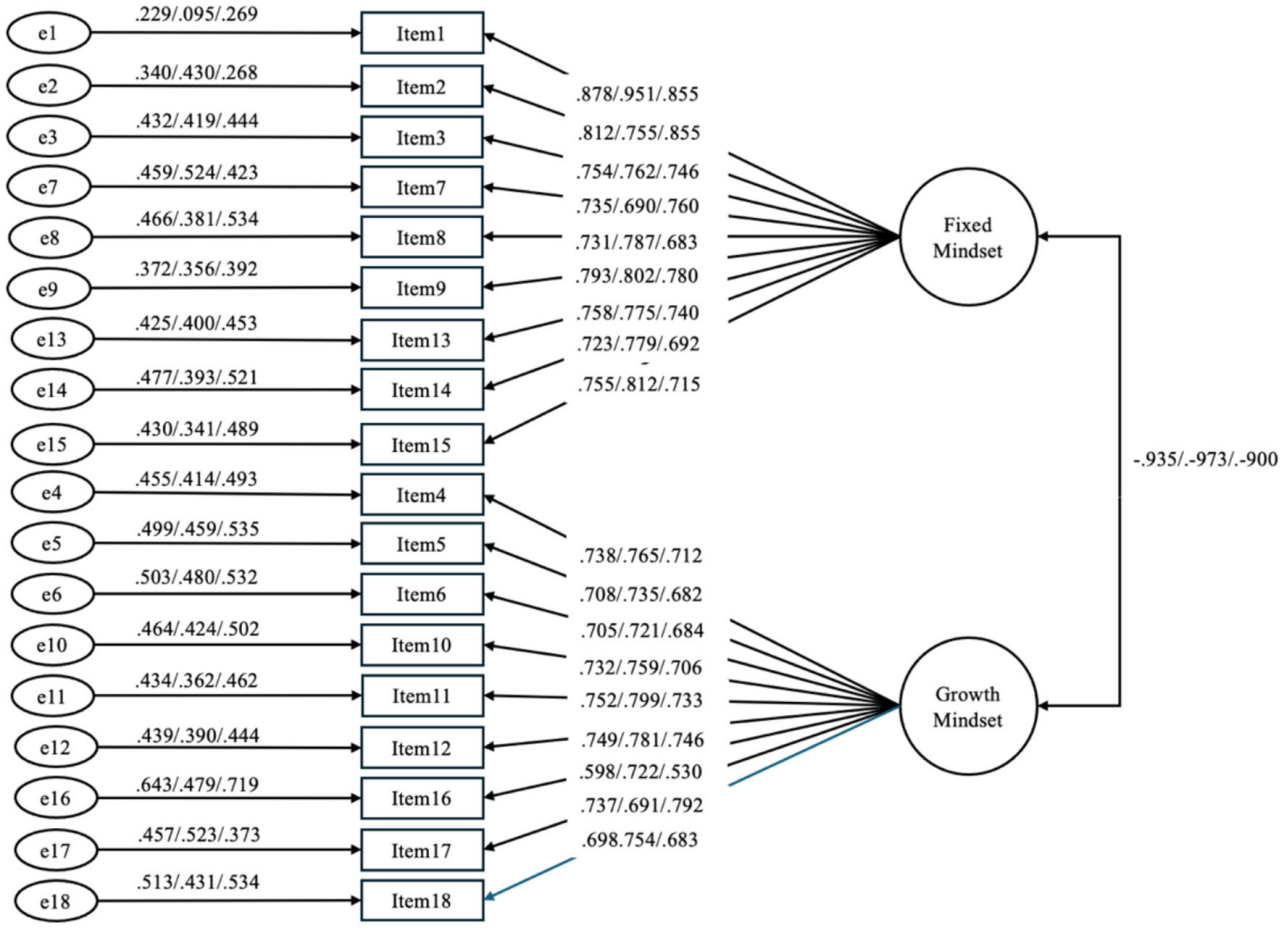
Standardized parameter estimates for the two-factor structure of the LMI among Chinese University Students.

The fit indexes for the one-factor model and the two-factor model utilizing CFA are presented in Table 1. Regarding total sample, in the one-factor model, the CFI, TLI, and REMSEA were slightly lower than the acceptable range. The standardized factor loadings of the one-factor model are depicted in Figure 1. In the two-factor model, the CFI, RMSEA, and SRMR were in the satisfactory ranges in spite of the TLI being slightly lower. Additionally, it could be observed that the male sample exhibited better fit indices in both one-factor model and two-factor model. Regarding female sample, the CFI value and TLI values in both models were close to 0.9, while RMSEA values were suboptimal in both model, which may be attributed to small sample size (Kenny et al., 2015). These results suggest that the two-factor model demonstrated better across groups. Therefore, the two-factor model was used as the initial baseline models for the subsequent examinations of measurement invariance. As shown in Figure 2, the standardized loadings ranged from 0.723 to 0.878 for fixed dimensions and from 0.698 to 0.752 for growth dimensions in total sample. The correlations between factors ranged from 0.820 to 0.945.

**Table 1:** Model fit statistics for the CFA models.

**Group**	**Model**	***X*^2^ (df)**	**CFI**	**TLI**	**RMSEA**	**SRMR**
Total (*n* = 476)	One-factor model	776.085 (135)	0.89	0.875	0.1	0.053
	Two-factor model	704.444 (134)	0.902	0.888	0.095	0.052
Male (*n* = 184)	One-factor model	202.480 (135)	0.973	0.969	0.052	0.03
	Two-factor model	192.422 (134)	0.976	0.973	0.049	0.03
Female (*n* = 292)	One-factor model	732.191 (135)	0.831	0.808	0.123	0.071
	Two-factor model	662.218 (134)	0.85	0.829	0.116	0.071

X^2^, chi-square; df, degrees of freedom; CFI, comparative fit index; TLI, Tucker–Lewis Index; RMSEA, root-mean-square error of approximation; SRMR, standardized root means square residual.

### External relation

As depicted in Table 2, the growth mindset dimensions of LMI were significantly positively associated with the 8-Item Growth Mindset Scale (*p* < 0.01) and the Mindset Scale for Learning English (*p* < 0.01). In contrast, the fixed dimensions of LMI were significantly negatively associated with both of them (*p* < 0.01).

**Table 2:** Intercorrelations among LMI, MATLE and GMS.

	**1**	**2**	**3**	**4**
1. MSLE	1			
2. GMS	0.167^**^	1		
3. Growth mindset	0.149^**^	0.186^**^	1	
4. Fixed mindset	−0.169^**^	−0.228^**^	−0.862^**^	1

^**^p < 0.01.

### Cronbach's alpha and mean inter-item correlation

Table 3 shows Cronbach's alphas and mean inter-item correlations for the LMI and its two dimensions for the total sample. The overall Cronbach's α value for the full scale was 0.954. Cronbach's α values for the two dimensions were 0.929 for the fixed dimension and 0.903 for the growth dimension. The results supported a strong internal consistency reliability of the scale. Additionally, the AVE values for two dimensions were 0.597 and 0.510 respectively, indicating an acceptable level. The CR values for the two dimensions were 0.930 and 0.903 respectively.

**Table 3:** Cronbach's alpha and mean inter-item correlation.

	**Full scale**		**Fixed**		**Growth**	
	α	**MIC**	α	**MIC**	α	**MIC**
Total	0.954	0.533	0.929	0.592	0.903	0.503
Male	0.962	0.583	0.936	0.624	0.919	0.558
Female	0.948	0.504	0.924	0.573	0.893	0.482

α, Cronbach's alpha; MIC, mean inter-item correlation.

## Discussion

In this study evaluating the LMI among Chinese university students, we investigated the suitability of one-factor and two-factor models, drawing on the fundamental approaches outlined by Dweck (2006) and enriched by perspectives from Lou and Noels (2017). This study focus was on evaluating the models' internal consistency and the fit indices from confirmatory factor analysis (CFA) to ascertain how well each model captured the constructs of fixed and growth mindsets.

In the examination of LMI among Chinese university students, the confirmatory factor analysis (CFA) for both the one-factor and two-factor models revealed significant insights. The Comparative Fit Index (CFI) and Tucker Lewis Index (TLI) for the two-factor model were 0.902 and 0.888, respectively, which are considered satisfactory in educational research settings. Similarly, the CFI and TLI for the one-factor model were slightly below the acceptable threshold at 0.89 and 0.875, respectively. Although the Root Mean Square Error of Approximation (RMSEA) for both models indicated values slightly above the ideal, which has shown 0.095 for the two-factor model and 0.1 for the one-factor model. However, these still fall within the acceptable limits, aligning with the standards suggested by Browne and Cudeck (1992), which consider RMSEA values below 0.10 as adequate for studies. This confirms the structural robustness of the LMI in capturing the distinct dimensions of fixed and growth mindsets.

In the study assessing the LMI among Chinese university students, the two-factor model emerged as more appropriate, effectively capturing the distinction between fixed and growth mindsets. Additionally, the items 16 (“Everyone could do well in foreign language if they try hard, whether they are young or old”) and 18 (“Regardless of the age at which they start, people can learner another language well”) displayed lower factor loadings. This could reflect the unique perspectives of our sample of university students, who may not perceive age as a significant barrier to language learning given their current life stage focused on academic and personal development (Stoten, 2015). Interestingly, this phenomenon finds resonance in the findings from Ryan and Mercer (2012), who noted that while discussing age-sensitive beliefs, fewer language learners mentioned age as a constraint. Furthermore, those who did often held fixed beliefs, suggesting that individuals with a growth mindset may be less likely to consider age as a limitation in their language learning capability (Lou and Noels, 2017).

Additionally, this study confirmed the correlations among MATLE, GMS, growth mindset, and fixed mindset, enhancing our understanding of these constructs within the theoretical framework. Notably, the correlation between MATLE and Fixed Mindset was relatively weak (*r* = −0.169) compared to its correlation with growth mindset (*r* = 0.149) and GMS (*r* = 0.167). This variation in correlation strength might be explained by the multifaceted nature of the mindset scales, namely, the LMI includes specific subdimensions such as age sensitivity beliefs about language learning (ASB), general language intelligence beliefs (GLB), and second language aptitude beliefs (L2B) (Horwitz, 1988; Lou and Noels, 2017). These subdimensions provide a more detailed framework for understanding cognitive and emotional responses in learning contexts, compared to the broader constructs of MATLE and GMS, which are more singularly focused.

The reliability of the LMI was thoroughly assessed using Cronbach's alpha, Average Variance Extracted (AVE), and Composite Reliability (CR). The full scale demonstrated excellent internal consistency with a Cronbach's alpha of 0.954. The fixed and growth mindset dimensions also exhibited strong internal consistency with alphas of 0.929 and 0.903, respectively. The AVE values for the fixed and growth dimensions were 0.597 and 0.510, respectively, both above the threshold of 0.5, indicating that a significant portion of the variance in observations is attributed to the underlying constructs (dos Santos and Cirillo, 2023). The CR values were also robust at 0.930 for the fixed dimension and 0.903 for the growth dimension, further affirming the scale's reliability. Overall, these metrics confirm the LMI scale's strong internal consistency and reliability in assessing mindset orientations within educational psychology.

Finally, this study results support the applicability of the two-factor model in assessing learning motivation among Chinese adolescents and indicate that the LMI is reliable for cross-gender comparisons. The suboptimal RMSEA values for the female sample may be attributed to the relatively small sample size (Hair et al., 2019; Kenny et al., 2015). Future research should aim to increase the sample size and test the measurement invariance of the LMI across different cultural contexts to further validate these findings.

### Implication

The validation of the LMI among Chinese university students provides an important tool for assessing and influencing the language mindset, which is also crucial for optimizing English language education. According to Collett and Berg (2020), the LMI accurately measures complex beliefs about language proficiency that have a significant impact on student engagement and persistence. This accurate assessment allows educators to identify harmful fixed-type mindsets and actively foster growth mindset through customized educational strategies, thereby increasing student motivation and resilience in language learning. Lou and Noels (2017) further suggest that integrating mindset assessment into regular educational practices not only promotes growth mindset, but also helps educators to systematically address cultural misconceptions about learning competence.

In addition, using LMI's continuous assessment feature, educators can dynamically adjust their teaching methods based on real-time feedback about changes in students' mindsets. This approach is critical in the Chinese educational setting, as combining mindset theory with traditional educational values can significantly improve learning outcomes. Research by Zarrinabadi and Lou (2022) suggests that the consistent use of LMI in the classroom helps to maintain the effectiveness of the intervention by ensuring that it is culturally relevant and meets the changing needs of students. Additionally, several studies support the broader application of growth mindset principles in educational settings, demonstrating their positive impact on student resilience and engagement in diverse cultural contexts (Meierdirk and Fleischer, 2022; Nieuwenhuis et al., 2023).

LMI can provide real benefits for teachers and students in educational settings. The assessment provided by the Language Mindset Inventory allows educators to adjust their teaching methods based on real-time feedback on students' mindset and improve students' growth mindset, which can lead to increased student engagement, motivation, and language proficiency (Lou and Noels, 2017). Training educators to understand and use mindset theories and measurements can improve their support for students' language learning journeys. Second, from a policy perspective, it is critical to raise educators' awareness of language mindsets. Educational authorities should integrate mindset theory into teacher training curricula. This integration would ensure that all educators are equipped with the knowledge and strategies to foster growth mindsets in their students. Professional development programs that emphasize the impact of fixed and growth mindsets on student achievement should be prioritized. Acceptable professional development contributes to successful implementation and positive change in instructional practices (Renko et al., 2021). Providing teachers with the tools to foster a growth mindset creates a more supportive and adaptive learning environment that fosters a resilient and motivated student body.

### Limitation and future study

Although this study makes an important contribution to understanding the language mindsets of Chinese university students, it recognizes a number of limitations. First, the generalizability of the findings is limited by the sample, which consisted of university students from mainland China. This particular population may not be fully representative of other potential groups of learners, such as younger students or adults outside the university, who may exhibit different language mindsets. To address this limitation, future research should include a more diverse sample that encompasses different age groups, educational backgrounds, and language proficiency levels. This will provide a broader understanding of the LMI applicability. Furthermore, although this study effectively validated the LMI, it did not explore the predictive validity of the LMI for actual language learning outcomes (e.g., grades, motivation, and attitudes). This limitation highlights the need for future research to investigate the correlations between the language mindset orientation measured by the LMI and other language learning related factors. Furthermore, the present study relied on the collection of self-reported data, and although this methodology is widely accepted and validated in psychological and educational research (Podsakoff et al., 2003; Chan, 2010; Conway and Lance, 2010), it may introduce biases such as socially expected responses or lack of self-awareness. These potential biases may limit the full understanding of Chinese university students' linguistic thinking. Future studies should consider using mixed methods, including indirect measures and multiple reports, to improve the robustness of the findings and reduce potential biases. Moreover, the cross-sectional design limits our understanding of the dynamic nature of language mindset. This design does not allow for tracking mindsets over time or understanding the trajectory of these mindsets. To address this issue, future research should adopt a longitudinal methodology to explore how linguistic mindsets develop and change in response to educational interventions, changes in the educational environment, or as students mature and encounter different academic challenges.

## Conclusion

This study validated the LMI among Chinese university students, providing a reliable measure for assessing language learning mindset in critical educational contexts. The confirmation of the two-factor structure of the LMI deepens our understanding of fixed and growth mindset, providing educators with a nuanced tool for identifying and modifying negatively charged language learning beliefs. The findings encourage the adoption of mindset-driven educational strategies that cater to learners' individual circumstances and promote a more personalized learning experience. There is a need to further explore the longitudinal impact of these mindsets and their broader applicability across cultures and educational settings in order to realize the full potential of LMI in promoting lasting educational enhancement.

## Data Availability

The raw data supporting the conclusions of this article will be made available by the authors, without undue reservation.
